# New feed sources key to ambitious climate targets

**DOI:** 10.1186/s13021-015-0040-7

**Published:** 2015-12-01

**Authors:** Brian J. Walsh, Felicjan Rydzak, Amanda Palazzo, Florian Kraxner, Mario Herrero, Peer M. Schenk, Philippe Ciais, Ivan A. Janssens, Josep Peñuelas, Anneliese Niederl-Schmidinger, Michael Obersteiner

**Affiliations:** 1 Ecosystems Services and Management, International Institute for Applied Systems Analysis, Schlossplatz 1, Laxenburg, Austria; 2 Commonwealth Scientific and Industrial Research Organisation, Brisbane, Australia; 3 Algae Biotechnology Laboratory, School of Agriculture and Food Sciences, The University of Queensland, Brisbane, Australia; 4 Laboratoire des Sciences du Climat et de L’Environnement, CEA-CNRS-UVSQ, Gif-sur-Yvette, France; 5 University of Antwerp, Wilrijk, Belgium; 6 CSIC, Global Ecology Unit CREAF-CSIC-UAB, Cerdanyola del Valles (Catalonia), Spain; 7 CREAF, Cerdanyola del Valles (Catalonia), Spain

**Keywords:** Protein, Livestock, Biofuels, Climate change, Food security, BECCS, CCS, FeliX

## Abstract

Net carbon sinks capable of avoiding dangerous perturbation of the climate system and preventing ocean acidification have been identified, but they are likely to be limited by resource constraints (Nature 463:747–756, 2010). Land scarcity already creates tension between food security and bioenergy production, and this competition is likely to intensify as populations and the effects of climate change expand. Despite research into microalgae as a next-generation energy source, the land-sparing consequences of alternative sources of livestock feed have been overlooked. Here we use the FeliX model to quantify emissions pathways when microalgae is used as a feedstock to free up to 2 billion hectares of land currently used for pasture and feed crops. Forest plantations established on these areas can conceivably meet 50 % of global primary energy demand, resulting in emissions mitigation from the energy and LULUC sectors of up to 544 $\pm$ 107 PgC by 2100. Further emissions reductions from carbon capture and sequestration (CCS) technology can reduce global atmospheric carbon concentrations close to preindustrial levels by the end of the present century. Though previously thought unattainable, carbon sinks and climate change mitigation of this magnitude are well within the bounds of technological feasibility.

**Electronic supplementary material:**

The online version of this article (doi:10.1186/s13021-015-0040-7) contains supplementary material, which is available to authorized users.

## Background

Recent assessments from the Intergovernmental Panel on Climate Change (IPCC) conclude that net anthropogenic carbon emissions must be eliminated before the end of this century in order to limit increases in mean global surface temperatures to less than 2 $^{\circ }$C [1, 2]. However, 1 PgC has been suggested as a floor for annual agricultural emissions, even if significant progress is made to close yield gaps [3]. In particular, livestock production currently accounts for roughly one fifth of total anthropogenic greenhouse gas emissions, a footprint which must be expected to grow as meat demand scales with the size and affluence of global populations [4, 5]. Given a dwindling global carbon budget, a carbon-negative energy sector stands out as an obvious target for offsetting sustained or growing emissions from other sectors.

Among carbon-sequestering technologies, photosynthesis is simultaneously the cheapest and most efficient known solution. Reductions in atmospheric CO$_2$ through increased biomass energy production coupled with carbon capture and underground storage (CCS) on a permanent or semi-permanent basis are theoretically possible and technologically foreseeable [6, 7]. However, large-scale biomass production for energy risks inflating food prices and land use change, creating a deep carbon debt which mitigates potential emissions benefits [8, 9]. Large-scale algaculture, an emerging technology, represents a potential source of biomass for energy production while avoiding these tradeoffs. More promisingly, however, microalgae can also be exploited as a source of animal feedstock, offsetting anticipated growth in demand for meat and dairy while allowing vast areas of agricultural land to be repurposed for biomass production or habitat restoration.

### Algaculture

Phototrophic microalgae production systems can potentially generate enough biomass to satisfy a large fraction of future global energy demand without shifting burdens onto forest or agricultural systems. In general, algacultural systems share several attractive characteristics which drive their effects on planetary boundaries. At present, productivities up to 100 dry biomass tons (tDM) ha$^{-1}$ year$^{-1}$ are seen as feasible in the southern United States, and output in excess of 150 tDM ha$^{-1}$ year$^{-1}$ has been achieved in closed production systems in climates as diverse as those of Benelux and the Maghreb [10, 11]. In the highest productivity systems, productivities are limited not by climactic conditions, but by carbon availability [11]. Microalgal strains and growing conditions can be selected or engineered to match varying climatic conditions and commodity demands, resulting in algal biomass highly enriched in proteins, carbohydrates, or lipids (typically 30 % and up to 60–75 % by dry weight), depending on its use [12].

Algacultural production systems can be constructed on degraded or otherwise unproductive land unsuitable for conventional feedstocks [13], mitigating competition for arable land as well as tradeoffs between bioenergy production and food security. Many strains grow in brackish or seawater, and freshwater can be recycled through many harvests, thus minimizing the impact of production systems on fresh water use [12, 14]. Nitrogen and phosphorous, both essential inputs, can be supplied partially with wastewater and excess manure stocks, thus closing crucial fertilizer cycles [13]. Finally, CO$_2$ can be supplied from flue gases or drawn directly from the atmosphere in open systems, making algae a nearly carbon-neutral commodity [12].

Protein-rich biomass is in all cases a co-product and in some cases the main product of these systems. As feedstock, studies have shown a range of species of eukaryotic microalgae to be equal or superior to conventional sources of carbohydrates and proteins in terms of nutritional value and digestibility [15, 16]. Net protein utilization, a compound measure of the digestibility and biological value of the protein contained in foods, varies from 20–40 %, and is on par with conventional sources [17]. Relative to conventional feeds, field studies have established neutral-to-positive effects on feed palatability, overall livestock growth and mortality rates, and meat taste for diets containing up to 10, 33, and 45 % microalgae for poultry, pigs, and ruminants, respectively [16, 18, 19]. As a result, microalgae represents a potential replacement for soy, fishmeal, and other conventional sources of protein. Pilot microalgae production systems have already been established (e.g. the Algae Energy Farm (AEF) in Queensland, Australia and the Kona Demonstration Facility in Hawaii), indicating that algal biomass for feed and fuel may soon become a commercial reality [20].

## Scenario definitions

We use the FeliX model to assess the emissions and land use consequences of maximal land-based biomass production for energy [21, 22]. In the business-as-usual (*BAU*) scenario, future global population growth, dietary patterns, energy profiles, and agricultural yields are assumed to develop along historical trends and without perturbation from policy changes or transformational technologies. In the *BioEnergy* scenario, the expansion of biomass, wind, and solar energy is accelerated exogenously to match more aggressive climate action scenarios [23], simulating an accelerated transition to renewable energies. Together, these scenarios define a baseline against which the impacts of alternative technologies can be measured. The construction and calibration of the *BAU* scenario are presented and discussed in depth in this paper’s SI and on the model website [21].

Relative to this baseline, we assess the maximum theoretical emissions mitigation potential of microalgae alternately as a source of biomass for energy (*Alg-Fuel*) and as a feedstock (*Alg-Feed*). In these scenarios, we impose exogenously the construction of 25-50 Mha of algaculture at a constant rate between 2015 and 2060. The output of these systems (up to 75 tDM ha$^{-1}$ year$^{-1}$) generates an annual total of up to 3.75 Gt DM [10]. On-shore microalgae production systems are assumed to occupy non-arable or permanently fallow land and to use recycled fresh or brackish water. Essential nutrients are supplied from municipal wastewater, agricultural runoff, or manure. Algae can be near carbon-neutral (modulo production and transportation costs), as CO$_2$ is sourced initially from flue gases from the combustion of fossil fuels and subsequently from bioenergy production. Other potential sources include steel and cement manufacturing facilities, small industries, agriculture, and open-air respiration [11]. To minimize the infrastructure, transportation, and emissions costs, proximity to essential inputs should be a primary consideration in the construction of algae farms [11, 13].

In the *Alg-Fuel* scenario, algacultural output is burned directly for electricity or used to produce biodiesel. In the *Alg-Feed* scenario, this output is instead used to meet 40 % of global demand for feed. Extrapolating from FAO feed demand estimates and assuming conventional plant-to-animal conversion efficiencies, the maximum required annual output (40 % of feed) ranges from 0.5 GtDM in 2010 to 1.0 GtDM in 2100 [24]. Thus, this scenario requires up to 25 Mha, allowing for a 50 % reduction in areal productivity (32.5 tDM ha$^{-1}$ year$^{-1}$) due to feed quality standards.

In all scenarios, biomass for bioenergy is sourced from the conversion of natural forests to plantations (10 tDM ha$^{-1}$ year$^{-1}$) or the use of agricultural land for energy crops (20 tDM ha$^{-1}$ year$^{-1}$) [25]. In all scenarios except *BAU*, agricultural residues from half of arable land are also collected for fuel (5.5 tDM ha$^{-1}$ year$^{-1}$). Areal yields consistent with highly-productive energy crops on prime agricultural land are applied irrespective of regional or climatic considerations in order to establish an upper bound on the impact of agricultural bioenergy production (cf. Table 1).

**Table 1:** Biomass streams for bioenergy production in the four main scenarios in this analysis

Bioenergy source (yield [tDM ha$^{-1}$ year$^{-1}$])	Scenario				Ref.
	*BAU*	*BioEnergy*	*Alg-Fuel*	*Alg-feed*	
Forest plantations (10)	$\checkmark$	$\checkmark$	$\checkmark$	$\checkmark$	[25]
Energy crops (20)	$\checkmark$	$\checkmark$	$\checkmark$	$\checkmark$	[25]
Agricultural residues (5.5)		$\checkmark$	$\checkmark$	$\checkmark$	[33]
Microalgae (*as fuel* 75)			$\checkmark$		[11]

In the *Alg-Feed* scenario, algal biomass is used as feedstock, not for energy production. Biomass from all sources is assumed to generate energy (17.45 GJ tDM$^{-1}$) and net emissions (0.049 tC tDM$^{-1}$, excluding land use change) uniformly

Each of these bioenergy production scenarios is evaluated over a range of energy sector emissions mitigation with CCS (25–75 %) reduction on emissions from the energy sector) to identify the CCS threshold at which each energy mix achieves net-zero anthropogenic emissions.

Leading systematic errors are determined from additional scenarios in which major model parameters are varied independently within an envelope of plausibility. We subsequently assess the impact of each parameter on emissions and warming projections. Many systematic errors in model results are correlated over all scenarios, and leading uncorrelated errors are quantified (Table 2), resulting in a robust assessment of scenario impacts relative to *BAU*.

**Table 2:** Dependence of cumulative (2011–2100) energy and land use sector emissions projections on critical model parameters

Cumulative emissions (2011–2100)		
Nominal $\big \vert \Delta$(*Alg-Feed*,*BAU*)$\big \vert$ = 544 $\pm$ 107 PgC		
Parameter shift	Down (Rel.)	Up (Rel.)
Agricultural emissions	539 (−0.9 %)	550 (1.0 %)
Agricultural residues	521 (−4.2 %)	565 (3.8 %)
Agricultural yields	540 (−2.6 %)	543 (−0.3 %)
Algae program start	–	494 (−9.2 %)
Biomass fixed emissions	602 (10.6 %)	487 (−10.5 %)
Energy crop productivity	572 (5.1 %)	538 (−1.2 %)
Energy demand	516 (−5.1 %)	569 (4.5 %)
Feed Pct. from algae	453 (−16.8 %)	–
Food demand (Ani.)	547 (0.5 %)	541 (−0.6 %)
Food demand (Veg.)	540 (−0.8 %)	548 (0.8 %)
Forest C sequestration	517 (−5.0 %)	571 (5.0 %)
Global GDP	537 (−1.2 %)	549 (0.9 %)
Non-CO$_2$ emissions	544 (0.0 %)	544 (0.0 %)
Plantation productivity	507 (−6.8 %)	578 (6.3 %)
World population	530 (−2.5 %)	554 (1.9 %)
Total error (uncorrelated, symmetrized): $\pm$19.6 %		

Cumulative emissions from the energy and land use sectors in the *BAU* and *Alg-Feed* scenarios are recalculated with each model parameter shifted independently as discussed in the SI. Emissions abatement for *Alg-Feed* relative to *BAU* is reported in absolute terms and as a percentage of the nominal value (544 PgC). Positive percentages indicate greater *Alg-Feed* scenario impact relative to *BAU* (additional climate mitigation), and negative values indicate diminished impact. Nominal parameters values and shift magnitudes are defined in this paper’s SI. All errors are assumed to be uncorrelated and summed quadratically in the bottom row

## Results

Demographics in the FeliX model are calibrated to leading exogenous projections. Global population is projected to reach 10.5 billion—a nearly 50 % increase—by 2100 [26], while global GDP per capita is projected to increase fivefold over the same period [27]. This growth leads to increases in per capita demand for energy (+25 %) and vegetal (+20 %) and animal (+40 %) food calories, all of which pose significant, well-documented challenges for global human and natural systems [3]. Within this context, the potential consequences of algaculture for fuel and feed can be evaluated in terms of associated land use changes and carbon emissions pathways.

### Energy profile and supply

**Fig. 1:**
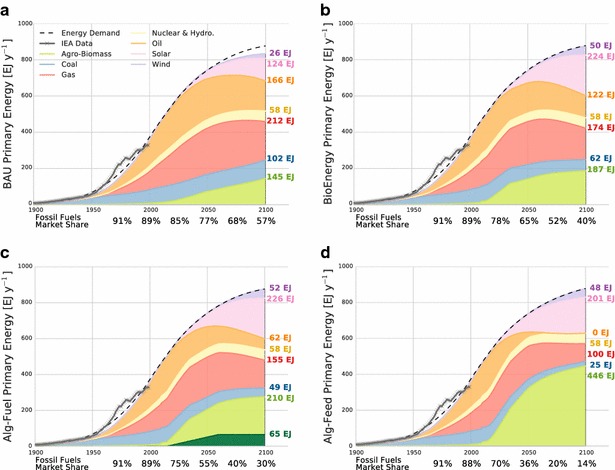
Total annual primary energy production [EJ] through 2100 in the **a** *BAU*, **b** *BioEnergy*, **c** *Alg-Fuel*, and **d** *Alg-Feed* scenarios. *Grey and black curves* indicate historical data and projected demand, respectively. *Colored numbers* on the *right* display the primary supply of each energy source in EJ. Historical data from IEA used for calibration [39]

Figure 1 displays the time series of primary energy production for the *BAU*, *BioEnergy*, *Alg-Fuel*, and *Alg-Feed* scenarios. Primary energy production in the *BAU* and *BioEnergy* scenarios is calibrated to baseline and aggressive climate action estimates, respectively, from comprehensive analyses [7, 23]. As such, these are not model results, but rather define the scenarios and represent benchmarks to frame discussion of the benefits and costs associated with each approach to energy production. Overall, the market share of fossil fuels falls from 95 % at the beginning of the century to 60 % in *BAU* and nearly 40 % in the *BioEnergy* scenario. These scenarios delimit a range of energy futures consistent with the GEA and fall between RCPs 4.5 and 6.0 [23, 28].

In the *Alg-Fuel* scenario, the projected annual output of 50 Mha of algaculture generates 65 EJ year$^{-1}$ (Fig. 1c), primarily offsetting oil consumption. If algal biomass is instead used as feed (*Alg-Feed*), agro-biomass production expands to 446 EJ year$^{-1}$ (Fig. 1d), 90 % of which is due to the productivity of former pasture and feed-crop land. This eliminates all dependence on oil and offsets demand for coal and gas, reducing fossil fuel consumption to under 20 % of primary energy supply.

### Land use change

**Fig. 2:**
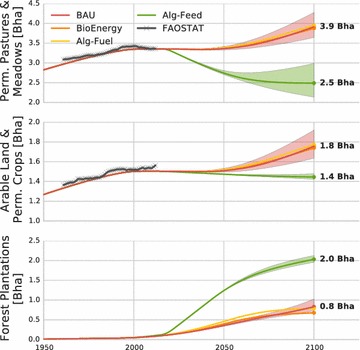
From *top*: time series of total extent of permanent pastures and meadows; arable land and permanent crops; and forest plantations. In the *Alg-Feed* scenario, microalgae is used to meet 40 % of demand for feed, and the land (1.8 Bha) spared is converted to plantation. *Shaded* ranges show the effects of population growth, the leading source of systematic error on agricultural land use projections. Historical data from FAOSTAT is used for model calibration [27]

In the FeliX model as in leading estimates, population and GDP growth combine to generate an 80 % increase in total calorie demand by 2100 [3]. This rate of growth in agricultural demand outpaces yield growth projections [29], driving expansion of arable land and permanent pastures and meadows by midcentury (Fig. 2) and increasing land use change emissions. Given this growth, agriculture for food and–increasingly–feed is likely to take up the most productive arable land, limiting the feasibility of large-scale first- and second-generation biofuel production on prime agricultural land [30]. Agricultural residues generate up to 50 EJ year$^{-1}$ in the *BioEnergy* scenario, less than a quarter of anticipated bioenergy demand.

Concurrent with agricultural expansion, the land needed to meet anticipated bioenergy demand grows to over 800 Mha in the *BAU*, *BioEnergy*, and *Alg-Fuel* scenarios. This must be expected to exacerbate competition for arable land, driving up food and energy prices and resulting in deforestation as biomass plantations replace natural and lightly-managed forests [30, 31].

Algaculture avoids such tradeoffs, simultaneously satisfying growing demand for animal products and biomass. Used as feed, microalgal biomass can free up to 40 % of pastures, meadows, and feedcrop land. This area—2.0 Bha in 2100 in the *BAU* scenario—is sufficient to produce annual biomass harvests equivalent to over 355 EJ. In this way, highly-productive algacultural technologies can be harnessed to offset the resource and environmental costs of rising demand for animal proteins.

### Emissions

**Fig. 3:**
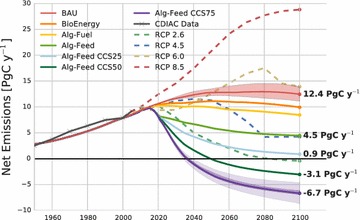
Net annual emissions. Defined as sum of carbon emissions from energy and land use sectors minus the carbon sequestered in biomass growth for bioenergy. *Dark shaded* ranges show the effects of population growth on the *BAU* and *Alg-Feed*+*CCS* projections, and the lighter range depicts sensitivity of the latter scenario to energy crop land productivity. For comparison to established emissions benchmarks, the four IPCC RCPs are also displayed [28]. Historical data from CDIAC is used for results validation [40]

**Fig. 4:**
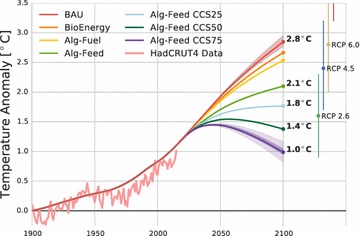
Global observed average temperature change relative to preindustrial levels. *Dark shaded* ranges show the effects of population growth on temperature change in the *BAU* and *Alg-Feed*+*CCS* scenarios. The *lighter-shaded* regions display the sensitivity of *BAU* results to alternative RCP scenarios and of *Alg-Feed* results to the biomass productivity of afforested land. For comparison to established emissions benchmarks, median IPCC temperature anomaly predictions in 2100 are shown at right with errors representing a 90 % confidence interval [1]. HadCRUT4 data are used for results validation, and represent observed temperature increases over preindustrial era from the Met Office Hadley Center

In the *BAU* scenario, annual emissions from the energy and land use sectors increase by 30 % between 2010 and 2100 despite growth in the total market share of renewable energies. This emissions pathway results in monotonically-increasing atmospheric CO$_2$ concentrations and a rise in global temperatures nearing 3 $\,^{\circ }$C above preindustrial levels by 2100 (Figs. 3 and 4). Marginal increases in biofuel production in the *BioEnergy* and *Alg-Fuel* scenarios do reduce carbon emissions, but do not represent the kind of transformation required to avoid 2 $\,^{\circ }$C warming–in part, because they do not reduce (and may increase) emissions from land use change.

More promisingly, significant expansion of renewable energies combines with avoided land use change emissions in the *Alg-Feed* scenario to achieve an emissions pathway between RCPs 2.6 and 4.5 [28]. Even before additional potential emissions benefits from CCS, this strategy is expected to reduce global temperature change 0.7$^{\circ }$C relative to *BAU* by 2100. Figures 3 and 4 project potential emissions and climate mitigation in the *Alg-Feed* scenario from the superposition of CCS (25, 50, or 75 % energy sector emissions reduction). With the addition of this technology, net zero emissions are achieved when at least 25 % of emissions from the energy sector are sequestered. Higher capture rates transform the energy and land use sectors into a net carbon sinks. The dependence of total emissions abatement in the *Alg-Feed* scenario (relative to *BAU*) on essential model parameters and other scenario-specific assumptions is listed in Table 2.

Though we have concentrated to this point on maximizing the emissions impact of algal feedstock by offsetting as much feed demand as possible, projected atmospheric carbon concentrations in year 2100 are shown for a range of algal contributions to animal feed (10–40 %) and energy sector emissions mitigation due to CCS (0–75 %) in Table 3. Yellow cells fall on the threshold for global surface warming (2.0 $\pm$ 0.2 $^{\circ }$C). Green cells indicate sub-2 $^{\circ }$C warming in year 2100 of the simulation, and red cells indicate supra-2 $^{\circ }$C change.

**Table 3:**
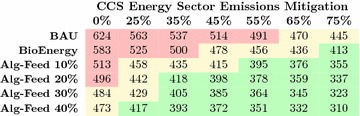
Projected atmospheric CO_2_ concentrations [ppm] in year 2100 of the simulation

A range of energy sector emissions mitigation from CCS (at top) are applied to a range of algae production scenarios (listed at left as percentages of total feed consumption). Yellow shaded cells indicate warming of 2.0 ± 0.2 $^{\circ }$C. Red shaded cells indicate warming projections in excess of this threshold, while green shaded cells indicate sub-1.8 $^{\circ }$C warming

Microalgal feedstock does not need to be maximized in order to contribute to the decarbonization of the energy and land use sectors. On any scale, algaculture creates the possibility of freeing large areas of arable land for biomass production. While the magnitude of the resulting carbon sink varies, microalgal feedstock holds significantly more promise than marginal improvements including conventional methods of agricultural intensification and algal biofuels, and is much closer to reality than other potentially transformational alternatives. For these reasons, algaculture and the land use optimization it enables should be seriously considered as essential tools for climate mitigation up to and including a decline in atmospheric carbon concentrations to preindustrial levels before the end of the century.

## Discussion and conclusions

The *BAU* scenario in this analysis forecasts increasing carbonization and warming of the atmosphere and oceans. This results in climate change, which will magnify demographic pressures on food and water security as well as forests and other ecosystems and functional biodiversity. In many ways, however, this baseline assumes the possibility of carrying on with business-as-usual through the end of the present century. Most importantly, it is possible that depletion of cheap fossil fuel reserves or growing appreciation of the threat posed by climate change will lead to greater-than-forecast demand for biomass. In the closed land system, failure to prepare for this transition will exacerbate competition for arable land, degrade food security, and accelerate deforestation and biodiversity loss.

In terms of emissions and temperature change, the *BioEnergy* and *Alg-Fuel* scenarios do not differ significantly from *BAU*. Ultimately, land scarcity precludes the possibility of establishing plantations and energy crops to produce enough biomass to substitute for a significant fraction of fossil fuel consumption [30, 32]. This leaves CCS (on scales that have not yet been proven feasible) or other so far unidentified technologies as alternatives for rapid, urgent emissions mitigation. Further, although the agricultural and plantation yields assumed in this analysis are conservative, harvesting of tree crowns and agricultural residues can deplete topsoils of vital nutrients, increasing dependence on fertilizers. In addition, an increase in demand for land-based biomass such as is foreseen in *BioEnergy* scenarios carries several hidden costs. In addition to land use change consequences, risks include disruption of ecosystems services including soil carbon sinks, biodiversity, and water cycles [7, 33, 34]. The direct and indirect costs of bioenergy production must be weighed against the benefits of this approach.

On the other hand, we have demonstrated the theoretical and technological potential of microalgal feedstock to relieve land scarcity, allowing arable land to be leveraged to produce cleaner energy while addressing the threats of climate change, deforestation, eutrophication, and food and water scarcity. In recognition of the fact that these systems can be engineered to produce biomass without generating a commensurate burden on critical ecosystems cycles and services, algacultural systems have already been established at some experimental farms to overcome biomass shortages in dry seasons.

The AEF currently produces algal biomass for feed at a cost of $1,840 per dry ton (cf. Additional file 1: Table S2). Prices as low as $500 per dry ton are generally seen as feasible, but only if CO$$_2$$ (57 % of AEF costs) can be sourced at no expense. At these prices, algae could have supplied 40 % of global feedstock in 2013 at a cost of 250–920 billion US$(2013). As a comparison, the global gross production value of livestock in the same year was 1,262 billion US$(2013), and fossil fuel subsidies totaled 550 billion US$(2013) [27, 35]. This estimate does not account for the value of algal co-products, nor does it include the land value created by the transformation of low-productivity pastures into plantations.

Algal biomass is already a viable alternative to fishmeal ($1,880 ton$$^{-1}$$) and should eventually compete with soymeal (average $375 ton$$^{-1}$$ over the last year) [36]. Critically, growth in demand for conventional protein sources has driven up the prices of both commodities (soy: 88 %, fishmeal: 165 % over the last decade), and this long-term trend can be expected to continue. On the other hand, research and investment in algacultural pilot programs will lead to higher productivities and lower costs for the cultivation, harvesting, and processing of algae at industrial scales. Looking forward, microalgae production systems represent an ideal transition technology from fossil fuels to bioenergy. For governments looking for shortcuts to sustainable development, algal feedstock manages to satisfy the competing imperatives of food security and climate mitigation by reducing resource burdens while commodifying CO$$_2$$. On large scales, this establishes the conditions for cascading greenhouse emissions savings and a return to preindustrial atmospheric carbon concentrations.

To be sure, there exist a number of challenges to engineering and operating algacultural systems on the scale envisioned in this analysis. Systems must be engineered which are robust against contaminant or mutant strains of algae, zooplankton, and viruses and other pathogens, which represent threats to stable and highly-productive monocultural systems [20]. As potential sites for colocation with carbon sources are exhausted, carbon capture and transportation infrastructures will need to be expanded at the same rate as algacultural production systems in order to maintain high productivities and low costs. Large scale carbon capture for algae production and permanent sequestration will likely increase energy consumption and costs, as will energy-intensive methods of algal biomass processing. For freshwater systems as well as those that fertilize with wastewater, the feasibility of recycling water through successive harvests in open and closed systems must be studied. Finally, the supply of nutrients to large scale algae production would necessitate an international market for manure and other forms or recycled nutrients from animal and human sources. Additional research should also be done to match leakage points in global nitrogen and phosphorous cycles with algacultural production systems, thereby minimizing the nutrient loads of these systems as well as the deleterious effects of agricultural runoff.

Apart from obstacles to large scale algae production, biodiversity loss–already a problem in the *BAU* and *BioEnergy* scenarios—would likely be exacerbated by even greater conversion of pasture and rangeland to energy crop plantations [37]. The elimination of pasture will require higher livestock densities, though rotational grazing in silvipastoral systems can mitigate animal crowding while fertilizing plantations, and the replacement of low quality feedstocks with algae may reduce reliance on antibiotics. Finally, afforestation and reforestation on the scale envisioned here would lower terrestrial albedo in these areas, potentially blunting the cooling impact of greater carbon sequestration [38]. Despite these unresolved questions, this analysis demonstrates that algacultural feedstock at any scale represents a promising and simultaneous solution to food security and climate change, and that these systems merit greater attention and closer scrutiny than they have thus far received.

## Additional file

**Additional file 1** The supplementary information for this paper contains descriptions of the FeliX model and the *BAU* scenario as well as a discussion of the model parameters shifted in the error analysis.

## Abbreviations

AEF: Algae Energy Farm at the University of Queensland in Queensland, Australia
BAU: business as usual
CCS: carbon capture and sequestration
IPCC: Intergovernmental Panel on Climate Change
tDM: metric ton dry biomass
