# Supplementary material for: New feed sources key to ambitious climate targets
Source: Carbon Balance Manag. 2015 Dec 1;10:26. doi: 10.1186/s13021-015-0040-7 (PMC4666903; doi:10.1186/s13021-015-0040-7)
Supplement: Supplementary file 1 — Additional file 1 The supplementary information for this paper contains descriptions of the FeliX model and the BAU scenario as well as a discussion of the model parameters shifted in the error analysis. [file 13021_2015_40_MOESM1_ESM.pdf]

RESEARCH

Supplementary Information for New feed sources key to ambitious climate targets

Brian J Walsh[1*], Felicjan Rydzak[1], Amanda Palazzo[1], Florian Kraxner[1], Mario Herrero[2], Peer M Schenk[3], Philippe Ciais[4], Ivan A Janssens[5], Josep Peñuelas[6,7], Anneliese Niederl-Schmidinger and Michael Obersteiner[1]

[*]Correspondence: walsh@iiasa.ac.at
[1]Ecosystems Services and Management, International Institute for Applied Systems Analysis, Schlossplatz 1, Laxenburg, Austria
Full list of author information is available at the end of the article

Contents

1 Systems Dynamics Modeling **5**

2 The FeliX Model **5**

2.1 Purpose . . . 5
2.2 Business as Usual (BAU) . . . 6
2.2.1 Population and GDP . . . 6
2.2.2 Food Demand . . . 7
2.2.3 Agricultural Yields . . . 8
2.2.4 Land Use . . . 8
2.2.5 Energy . . . 10
2.2.6 Carbon Cycling . . . 11
2.2.7 Climate . . . 12

3 The Algae Feed Scenario **13**

3.1 Algae Costs . . . 13
3.2 Agricultural Yields and Land Use . . . 13
3.3 Emissions . . . 15

4 Sensitivity Analysis **16**

5 Author Information, Acknowledgments, and References **18**

List of Figures

S1 **(A)** Global fertility rate and **(B)** life expectancy compared to historical data from the FAO. **(C)** Population in BAU, FAO data, and SSPs 1-3. In each plot, the red shaded range indicates the effect of the low and high boundaries of the 80% confidence interval on the WPP medium variant. **(D)** Historical data and FeliX model BAU figures for gross world product per capita. The red shaded range indicates the effect of lower and higher GDP growth scenarios. SSPs 1, 2, and 3 are also shown for comparison. 7

S2 **(Top left)** per capita animal and vegetal food demand; **(top right)** total animal and vegetal food production; **(middle left)** cropland yield (vegetal food) compared to global population; **(middle right)** permanent pastures & meadows, arable land & permanent crops, and forest plantation; **(bottom left)** agricultural, forest and other land; **(bottom right)** the same land use categories, stacked. 9

S3 At left: Energy demand per capita in BAU is a function of GPD per capita. Historical data is derived from energy and population data from the IEA and FAO, respectively. To assign a systematic error to this measurement, the nominal projection is shifted ±0.25% annually after 2010. This leads in 2100 to -20% lower and +25% higher energy demand per capita, as indicated by the red shaded region. At right: Total primary energy demand and production. 10

S4 **(A)** Annual primary energy production in the BAU scenario [EJ]. Historical data from the IEA is included for coal, oil, and gas production, and GEA scenario projections [1] for 2100. **(B)** Market shares of fossil fuels (left) and renewable energies (right), expressed as fractions of primary energy production. In all cases, shaded regions indicate the effect of alternative energy demand scenarios on primary energy consumption. 11

S5 Central diagram: carbon flow through atmospheric, biospheric, pedospheric, and oceanic reservoirs. At left: model formulas for calculating carbon flux, $\Sigma(X \rightarrow Y)$, [PgC y^{-1}] between reservoirs. Plots 1-5: parameter nominal values and annual carbon flux. 12

S6 **Top:** Gross annual emissions in tons C from fossil fuels, land use change, and renewable energies. **Bottom left:** Net annual emissions from the energy and LULUC sectors. The red shaded region indicates the effect of alternative population projections on emissions. **Bottom right:** Atmospheric CO_2 concentration. The red shaded region indicates the effect of alternative population pathways on emissions and atmospheric carbon concentration projections. 13

S7 Clockwise from top left: table of radiative forcings due to all GHGs in the *BAU* scenario. Forcings for all non-CO2 gases are nominally equal to RCP 4.5. Total radiative forcing in *BAU* scenario compared to all RCPs. Temperature change relative to preindustrial climate (°C). Historical data drawn from GISS and HadCrut4. In the plots, the darker shaded range indicates the effects of high and low population projections, and the larger, lighter range indicates the effect of switching all non-CO2 pathways to RCP 2.6 and RCP 8.5. 14

S8 (L) Time series of global cropland food and feed yields (shown for all scenarios) and global population (shown for *BAU* scenario). Independent, econometric projections of crop yields projections, displayed as dark grey bars, are used to validate model yield projections [2]. (R) Global agricultural, forest, and "other" land usage in billions of hectares. "Other" includes all land not classified as agricultural, forest, or urban/industrial. Shaded ranges show the effects of population growth, the leading source of error for the *BAU* and *Alg-Feed (40%)* agricultural land projections. Historical data from FAOSTAT used for calibration [3]. 15

S9 From top: time series of total extent of permanent pastures & meadows; arable land & permanent crops; and forest plantations. In the *Alg-Feed (40%)* scenario, microalgae is used to meet 40% of demand for feed. Of the 1.8 Bha agricultural land this frees, 1.4 Bha is converted to forest plantations. Shaded ranges show the effects of population growth, the leading source of systematic error on agricultural land use projections. Historical data from FAOSTAT used for calibration [3]. 16

S10 Time series of atmospheric CO_2 concentrations. Dark shaded ranges show the effects of population growth on the *BAU* and *Alg-Feed (40%)+CCS* projections, and the lighter range depicts sensitivity of the latter scenario to energy crop land productivity. For comparison, the four IPCC RCPs are also displayed [4]. Historical data from CDIAC used for validation [5]. 17

S11 **(Left)** Net annual emissions from the energy and land use sectors [GtC y^{-1}] in year 2100 for a range of emissions mitigation with CCS (gross energy sector emissions reductions) and levels of algae production (as percentage of total feed demand). **(Right)** Projected temperature anomalies relative to preindustrial [°C] in year 2100 of the FeliX simulation. In both tables, global temperature change of 2.0±0.2°C is indicated by the yellow cells. Green cells indicate sub-2°C warming in year 2100 of the simulation, and red cells indicate greater than 2°C warming. 17

List of Tables

S1 Dimensionless coefficients used to model the competing effects of agricultural yield intensification (INT and land management) and yield-limiting factors. The product of six coefficients, listed in the rightmost column, indicates the scaling factor used to calculate global average cropland yield (see Figure S2 at middle left). 8
S2 Marginal costs of algae production for feedstock at the Algae Energy Farm in Queensland, Australia. 14
S3 Emissions intensity and annual emissions for five major fuel sources and land use & land use change (LULUC) in the *BAU*, *BioEnergy*, *Alg-Fuel* and *Alg-Feed (40%)* scenarios in 2100. In the *Alg-Fuel* and *Alg-Feed (40%)* scenarios, total emissions are also tabulated after CCS is used to capture 25%, 50%, or 75% of annual emissions from the energy sector. Net emissions intensity is shown for biomass. Emissions intensity source: IIASA Global Energy Assessment 15
S4 Sensitivity analysis of leading model parameters affecting cumulative (2010-2100) emissions projections in the *BAU* and *Alg-Feed (40%)* scenarios. Absolute shifts listed in this table are used to calculate relative effects in Table 2 in the main paper. Parameters are shifted as described in relevant sections of this document. 17

1 Systems Dynamics Modeling

The system dynamics approach to modeling global impact modeling was originally developed by Jay Forrester at MIT in the 1950s [6, 7]. An alternative to reductionism, which dissects complex phenomena into component pieces, system dynamics attempts to comprehend entire systems to understand their past behavior and future evolution [8]. The foundational notion of this approach is that structure determines performance, i.e. that the structure of a system is the primary cause for its behavior–problematic or otherwise [9]. In practice, this means that systems dynamics models do not optimize objective functions to identify dynamically efficient pathways. By integrating historical data sets, FeliX generates insights into the effects of system constructions and resource allotments, but does not spontaneously or endogenously contemplate alternative systems or allotments.

In this context, "system" is defined as a collection of interrelated and interacting nodes, or elements, and "dynamics" refers to the temporal evolution of the system as governed by the same interactions. To maintain correspondence with reality, system dynamics models capture as many of the interactions among the elements within their scope as possible. In this way, they provide insight into feedback loops, or the co-dependent evolution of ostensibly separate sectors of dynamic systems. A change in one variable affects other variables in predictable ways over time. However, this effect can subsequently propagate to alter the course of the original variable, and so on. As these changes accumulate, insight can be gained into trends at the variable, sectoral, and global level. In general, these effects are linear, and complex models can be constructed out of a network of elementary interactions. However, special dynamic notions are also incorporated by delays and other nonlinear relations among the system elements. A thorough description of system dynamics components and technique is presented by [7, 8, 9, 10].

The system dynamics approach to characterizing and quantify the functioning of the climate system is well established [11, 12, 13, 14, 15, 16]. In cases where such relations have not been quantitatively established, group model building sessions have been convened [17, 18, 19, 20]. These foundational efforts provide both the philosophical and functional basis for the FeliX model, as described in this document.

2 The FeliX Model

FeliX models the effects of new policies and technologies in the context of fundamental, complex interconnections among social, economic, and environmental Earth subsystems in the Anthropocene Era [21, 22]. The model consists of differential equations which link stocks–representing resources–and variables to characterize the present state and future development of natural and economic systems, including: population, GDP, land use, energy, carbon cycling, and climate. Feedback loops define the connections among and drive the co-dependent evolution of these systems. All FeliX model historical data, parameters, and results are calculated and reported as global averages.

2.1 Purpose

The model combines historical data with results from similar models, impact figures from published articles and sector reports, and expert interviews to calibrate

the model to match available historical data between 1900 and 2010 [3, 23]. Future developments in the energy sectors are loosely calibrated to the *Global Energy Assessment* (GEA) [1]. Therefore, for the purposes of this analysis, primary energy profiles are not FeliX model results, but are rather definitional of scenarios.

This document describes the major features and linkages of the FeliX model with a focus on the nominal scenario, Business as Usual (*BAU*). Page numbers, where listed, refer to the model report and technical documentation [24], which contains a complete discussion of the representation and calibration of each sector, and which is available for download from the model website [25].

2.2 Business as Usual (*BAU*)

BAU seeks to project the development of agricultural, energy, and carbon systems in the absence of perturbation from new policies. Population growth, per capita food and energy consumption, agricultural yields, and land use evolve endogenously through 2100. In this way, *BAU* serves as a baseline against which the impacts of novel technologies and policies can be measured.

To understand sources of systematic error in *BAU* assumptions, we define an envelope of plausibility for eleven major model parameters around their nominal values. Parameters governing per capita demand for and supply of food, feed, and fuel are included as sources of error, and the shifts assigned to each parameter are defined in the relevant section of this report. Treating each parameter as uncorrelated, we run these through the model as independent scenarios. At the end of this report, we use these scenarios to derive estimates of the leading systematic errors of carbon emissions and temperature change projections in *BAU*. Finally, we use this method to examine leading errors on the emissions mitigation of an accelerated transition to renewable energies (*BioEnergy* scenario).

2.2.1 Population and GDP

Using a systems dynamics approach, population change is the net effect of fertility and mortality rates. The fertility rate is inversely correlated with educational attainment (p. 252) and GDP growth, and the mortality rate is derived from life expectancy, which is correlated directly with GDP growth and food availability and indirectly with environmental and climactic degradation.

Fertility, life expectancy, and population sub-groups are calibrated simultaneously to FAO data (Figure S1 A-C) (pp. 26-27). The *World Population Projection* Medium Variant and the Shared Socio-economic Pathways (SSPs) are used to validate the resulting baseline population projection (Figure S1 C) [26, 27].

The FeliX model uses a neoclassical growth model to characterize the economic sector (p. 11). World gross productivity (GDP) is correlated with labor force, capital accumulation, and the pace of technological developments and calibrated to historical data (Figure S1D) [28].

The population and GPD sectors of the FeliX model determine food, feed, and energy demand and are in turn affected by the aggregate effects of other model sectors on birth rates and life expectancies (p. 25).

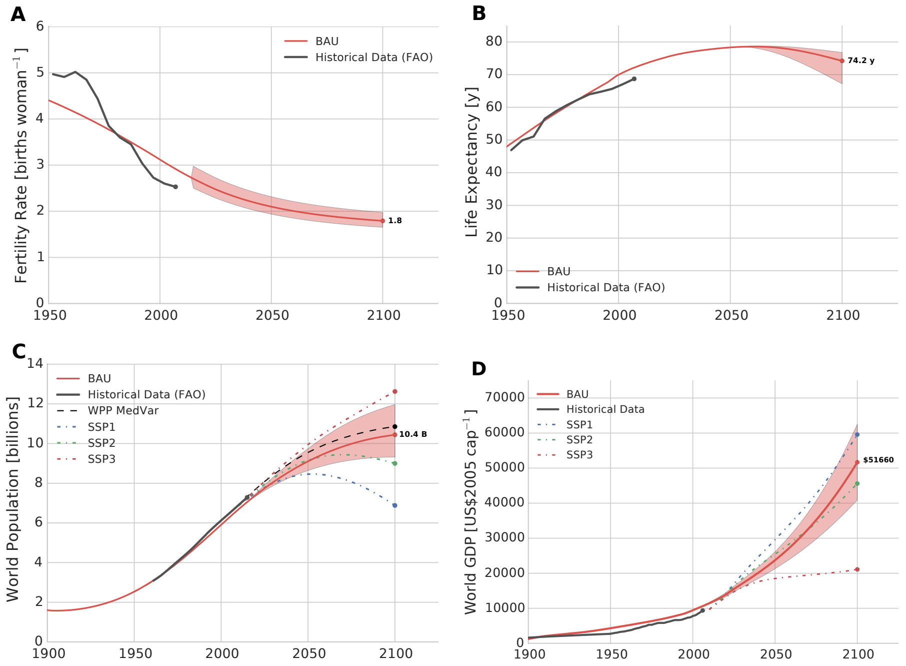

Figure S1 **(A)** Global fertility rate and **(B)** life expectancy compared to historical data from the FAO. **(C)** Population in *BAU*, FAO data, and SSPs 1-3. In each plot, the red shaded range indicates the effect of the low and high boundaries of the 80% confidence interval on the WPP medium variant. **(D)** Historical data and FeliX model *BAU* figures for gross world product per capita. The red shaded range indicates the effect of lower and higher GDP growth scenarios. SSPs 1, 2, and 3 are also shown for comparison.

Error Analysis

- **World Population:** The full width of the 80% confidence interval from the *World Population Projection* is used to define low and high population scenarios, as shown in Figure S1C.
- **Global GDP:** The baseline GDP growth projection is accelerated or retarded linearly between 2010 and 2100 to generate a total cumulative ±20% shift in GDP per capita (relative to nominal) by 2100, as shown in Figure S1D.

2.2.2 Food Demand

Per capita food demand is modeled separately for animal and vegetal calories and calibrated to historical data from the FAO (Figure S2 at top left). Future demand is scaled between lower limits (10 and 750 kCal day^{-1} for animal and vegetal, respectively) and upper limits (850 and 3,000 kCal day^{-1}) as a function of GDP per capita (pp. 262-263).

Error Analysis

- **Food Demand (Animal):** Nominal per capita animal food demand is scaled linearly between 2010 and 2100 to generate a cumulative ±10% shift by 2100, as shown in Figure S2 at top left.
- **Food Demand (Vegetal):** Nominal per capita vegetal food demand is scaled linearly between 2010 and 2100 to generate a cumulative ±10% shift by 2100, as shown in Figure S2 at top left.

Year	Dimensionless Productivity Factors (γ_i)						$\prod_i(\gamma_i)$
	INT	Management	Water	Pollution	C Fert.	Climate	
1900	1.00	1.10	0.99	1.00	1.00	1.00	**1.09**
2010	1.67	5.86	0.99	0.97	1.01	1.00	**9.53**
2050	1.88	7.96	0.98	0.94	1.03	0.99	**14.11**
2100	2.12	8.00	0.84	0.92	1.04	0.99	**13.43**

Table S1 Dimensionless coefficients used to model the competing effects of agricultural yield intensification (INT and land management) and yield-limiting factors. The product of six coefficients, listed in the rightmost column, indicates the scaling factor used to calculate global average cropland yield (see Figure S2 at middle left).

- **Feed Percentage from Algae:** A range of feed demand reduction due to algae production [10%, 20%, 30%, 40%] is considered in the main paper. For the purposes of this error analysis, 40% of feed demand is nominally met by algae in the *Alg-Feed (40%)* scenario, and a 50% shift down (to 20% of feed demand) is used in Table S4.

2.2.3 Agricultural Yields

The land use and land use change (LULUC) sector of the model accounts for agricultural intensification due to fertilization, irrigation, and input-neutral technological (INT) advancement, and for de-intensification due to water deficits, climate change, and pollution. Yield scale factors associated with each of these factors are modeled independently (pp. 66-68) and tabulated below.

Error Analysis

- **Agricultural Yields**: The pace of input-neutral yield growth–nominally a linear extension of historical trends (p. 265)–is shifted up or down to create a ±25% effect in areal agricultural yields, as shown in Figure S2 at middle left. This range is constructed to encompass independent econometric projections of input-neutral yield growth through 2100 [29], indicated by grey bars in the same figure.

2.2.4 Land Use

Agricultural production systems can either intensify of expand in response to growth in demand for feed, food (animal and vegetal) and fuel. In addition to land (pp. 58-74), water (pp. 93-99) input is explicitly modeled.

Four types of land are distinguished in the FeliX model: agricultural, forest, urban/industrial, and other (e.g. woodland and grassland). Agricultural land is further partitioned into arable land, permanent crops, and permanent meadows and pastures. Competition among these mutually-exclusive land categories is mediated by demand for the commodities they produce, as discussed above, and by policy restrictions. In particular, demand for food, feed, fuel, and fiber drive the expansion of agricultural land and managed forests at the expense of natural forests and other habitats.

The allocation of agricultural land is shown in Figure S2 (middle right), with total land area shown at bottom. The most prominent trend in this figure is the

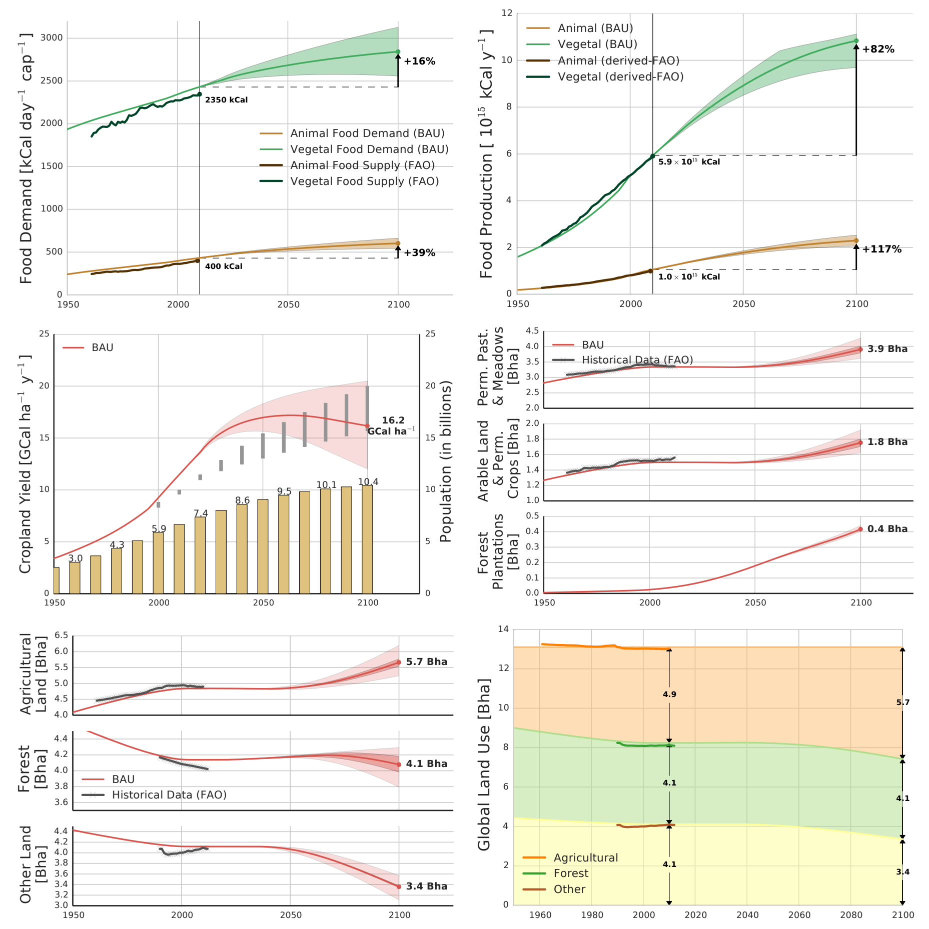

Figure S2 **(Top left)** per capita animal and vegetal food demand; **(top right)** total animal and vegetal food production; **(middle left)** cropland yield (vegetal food) compared to global population; **(middle right)** permanent pastures & meadows, arable land & permanent crops, and forest plantation; **(bottom left)** agricultural, forest and other land; **(bottom right)** the same land use categories, stacked.

expansion of agricultural land by over 33% at the expense of forest (16% decrease) and other natural habitats (27% decrease). This expansion is required to meet growing demand for food, feed, and energy as well as to offset anticipated decreases in the productivity of agricultural land due to pollution, exhaustion, and water scarcity.

Error Analysis

- **Plantation Productivity** The nominal productivity of managed forests (10 dry tons biomass $ha^{-1}y^{-1}$) is shifted $\pm 50\%$ to cover a range of 5-15 dry tons biomass $ha^{-1}y^{-1}$.
- **Energy Crop Productivity** The nominal productivity of energy crop land (20 dry tons biomass $ha^{-1}y^{-1}$) is shifted $\pm 50\%$ to cover a range of 10-30 dry tons biomass $ha^{-1}y^{-1}$.

2.2.5 Energy

Energy demand per capita is calculated as a function of GDP per capita (pp. 45-55) and plotted at left in Figure S3. Supply is modeled independently for each primary source of energy: coal, gas, oil, solar, wind, and biomass (Figure S4A). Nuclear power is limited exogenously in *BAU* to present production. The market share of each source is presented in Figure S4B with IEA historical data.

Middle-of-the-road GEA projections (*GEA_med_450* & *geama_450_btr_full*) for 2100 are indicated in brackets in Figure S4A [1]. Primary energy projections through 2100 are compatible with these moderate GEA pathways circa 2100 with respect to total primary energy production (GEA: 850-909 EJ y^{-1} vs. *BAU*: 875 EJ y^{-1}), total production minus nuclear and hydroelectric power (GEA: 679-830 EJ y^{-1} vs. *BAU*: 779 EJ y^{-1}), and fossil fuel usage (GEA: 122-539 EJ y^{-1} vs. *BAU*: 482 EJ y^{-1}). We also note that FeliX *BAU* does not include carbon capture and sequestration, while the GEA pathways do.

On top of this calibration, a simulation of price-based competition between energy sectors explicitly models sectoral and global growth and efficiency due to exploration, production, infrastructural investment, R&D activities, and costs of energy carriers. This module is used for impact analysis of exogenous market deformities including renewable resources subsidies and other policy tools.

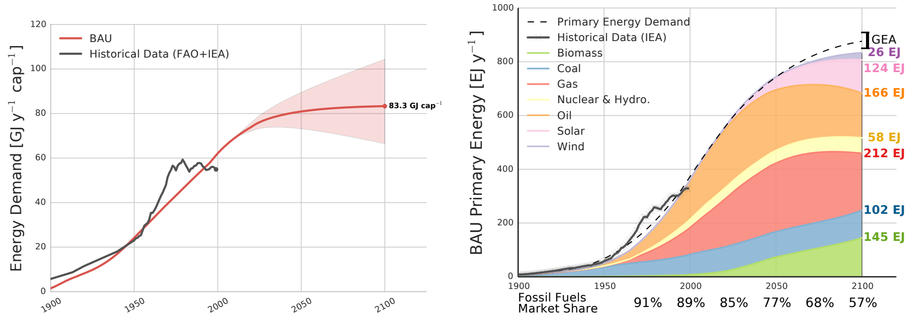

Figure S3 At left: Energy demand per capita in *BAU* is a function of GPD per capita. Historical data is derived from energy and population data from the IEA and FAO, respectively. To assign a systematic error to this measurement, the nominal projection is shifted ±0.25% annually after 2010. This leads in 2100 to -20% lower and +25% higher energy demand per capita, as indicated by the red shaded region. At right: Total primary energy demand and production.

As shown in Figs. S3 and S4, renewable sources of energy including solar, wind, and biomass are projected even in *BAU* to undergo significant expansion. As a result, the market share of fossil fuels is projected to fall below 60% of primary energy supply by 2100, even as absolute consumption rises 17% relative to 2010. Over the same period, biomass production grows to satisfy nearly a quarter of global energy demand.

In the *BioEnergy* scenario (cf. Fig. S4), absolute fossil fuel consumption falls 12% in 2100 relative to 2010. Over this period, the market share of renewable energies expands to 60% of total primary energy production.

Error Analysis

- **Energy Demand** Per capita energy demand is shifted by a factor of ±0.25% y^{-1} from 2010-2100 to simulate on the low side gains in energy use efficiency

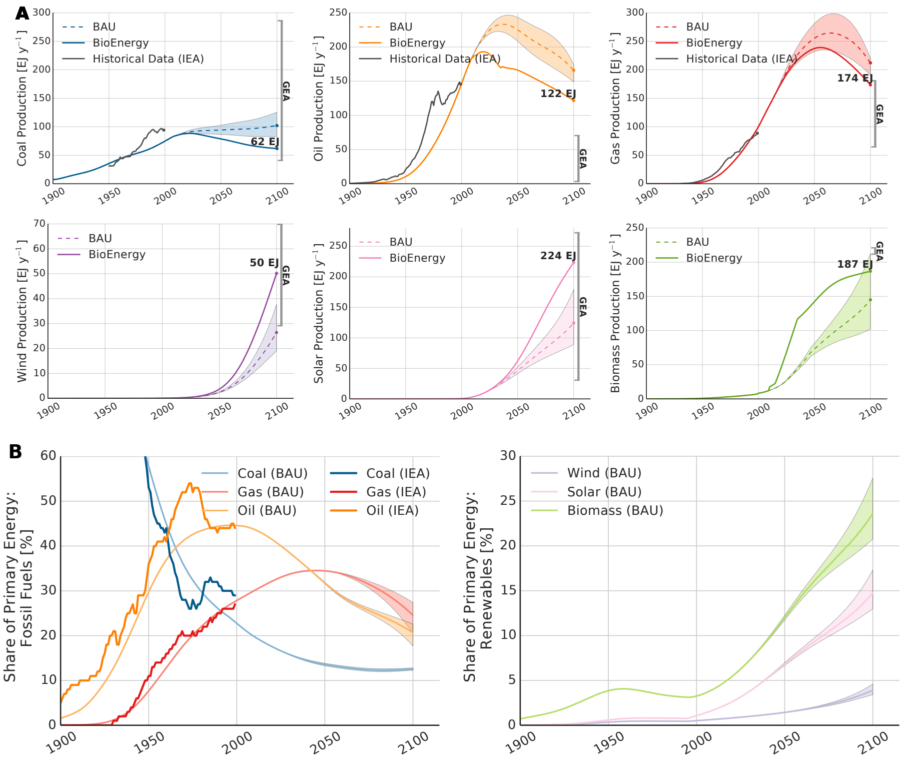

Figure S4 **(A)** Annual primary energy production in the *BAU* scenario [EJ]. Historical data from the IEA is included for coal, oil, and gas production, and GEA scenario projections [1] for 2100. **(B)** Market shares of fossil fuels (left) and renewable energies (right), expressed as fractions of primary energy production. In all cases, shaded regions indicate the effect of alternative energy demand scenarios on primary energy consumption.

and, on the high side, increased demand. This geometric approach shifts total energy demand asymmetrically, with low demand at 701 EJ y^{-1} (-20% relative to nominal) and high demand at 1091 EJ y^{-1} (+25%) in 2100.

2.2.6 Carbon Cycling

The climate sector of the FeliX model is based on the C-ROADS model [30], which in turn refers to the FREE [16, 31] and DICE [32, 33] models (pp. 76-82).

This sector for CO_2 emissions with a detailed representation of emissions in the energy sector and land-use change. Energy production technologies differ in their carbon intensities. The model accounts for CO_2 emissions from oil, gas, coal, biomass, solar, and wind energy technologies for their full life-cycle. Furthermore, the model uses the carbon cycle model proposed by [16], in which CO_2 emissions accumulate in the atmosphere until they are reabsorbed into the terrestrial biosphere and the ocean. The model also accounts for CO_2 flux between living biomass and humus and also distinguishes between the ocean's mixed layer and the deep ocean (pp. 86-91).

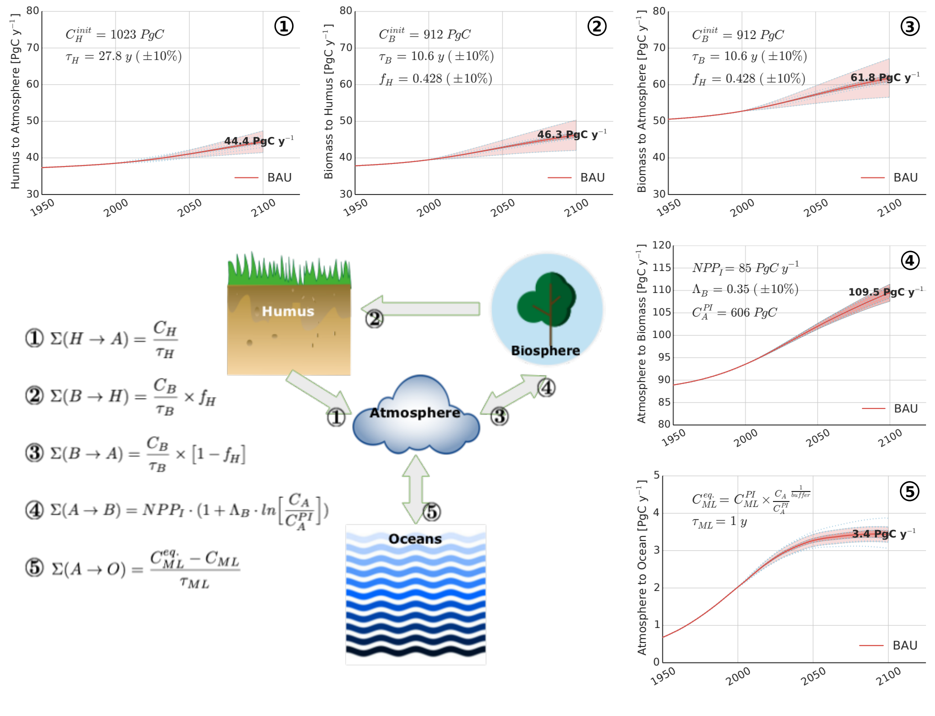

Figure S5 Central diagram: carbon flow through atmospheric, biospheric, pedospheric, and oceanic reservoirs. At left: model formulas for calculating carbon flux, Σ(X→Y), [PgC y^{-1}] between reservoirs. Plots 1-5: parameter nominal values and annual carbon flux.

Error Analysis

- **Biomass Fixed Emissions** Nominal net emissions from bioenergy (0.05 tC tDM^{-1}), which reflect transportation and processing energy costs, are shifted ±100%.
- **Agricultural Emissions** Nominal agricultural emissions (including those from fertilizer)–which total 1.12 PgC in 2010 and 2.16 PgC in 2100–are shifted ±20%.
- **Forest C Sequestration** The carbon sink of standing forests (nominally 85 tC ha^{-1}) is shifted ±25%. This affects the carbon penalty for deforestation as well as the incentive for afforestation.

2.2.7 Climate

The climate sector of FeliX draws out the effects on climate of the accumulation of carbon dioxide (CO_2), methane (CH_4), nitrous oxide (N_2O), hydrofluorocarbons (HFCs), and other greenhouse gases. Specifically, it simulates the warming on the surface of the Earth and in the upper ocean due to these emissions in accordance with [31] and [33]. The Representative Concentration Pathway projection of 4.5 W/m^2 is used for all radiative forcings except CO_2, which is determined endogenously. Positive forcing increases the atmospheric and upper ocean temperatures, as shown in Figure S7, and the transfer of carbon and heat into deeper ocean layers is also modeled explicitly (pp. 84-91). The consequences of climate change are subsequently propagated through all sectors, with consequences for land fertility, population growth, and biodiversity, among other parameters (p. 89).

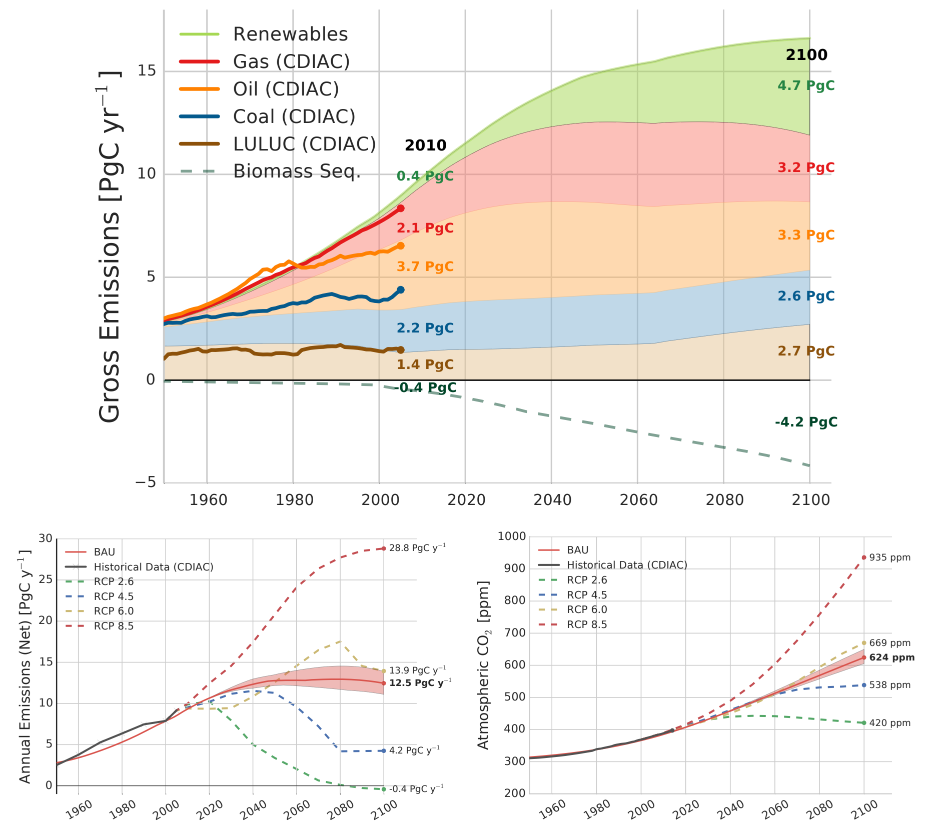

Figure S6 Top: Gross annual emissions in tons C from fossil fuels, land use change, and renewable energies. **Bottom left:** Net annual emissions from the energy and LULUC sectors. The red shaded region indicates the effect of alternative population projections on emissions. **Bottom right:** Atmospheric CO_2 concentration. The red shaded region indicates the effect of alternative population pathways on emissions and atmospheric carbon concentration projections.

Error Analysis

- **Non-CO_2 Emissions:** RCPs for CH_4, N_2O, HCFs, and "other" greenhouse gases are shifted down (RCP 2.6) or up (RCP 8.5) simultaneously to model the warming effects of low and high non-CO_2 emissions pathways, respectively.

3 The Algae Feed Scenario

3.1 Algae Costs

The operating costs of the Algae Energy Farm in Queensland Australia are listed in Table S2. Capital expenditures are assumed to be amortized over 20 years at 3% interest y^{-1}. Carbon dioxide is the single most expensive input, but co-location with industrial carbon streams and the expansion of CCS infrastructures would make it feasible for algae farms to be paid to consume these emissions.

3.2 Agricultural Yields and Land Use

Projected yields and avoided agricultural land use change in the *Alg-Feed (40%)* scenario (relative to *BAU*) are plotted in Fig. S8. Areal yields are higher in the *Alg-Feed (40%)* scenario due to reduced competition for water (irrigation), but this effect is smaller than the error envelope assumed for agricultural yields. Time series

	Radiative Forcing [W/m^2]					
	2000	2020	2040	2060	2080	2100
CO_2	1.49	2.06	2.69	3.30	3.85	4.35
CH_4	0.49	0.50	0.52	0.50	0.45	0.42
N_2O	0.15	0.19	0.23	0.27	0.30	0.32
HFC	0.33	0.35	0.32	0.24	0.20	0.18
Others	-0.78	-0.60	-0.43	-0.30	-0.23	-0.22
Total	1.67	2.50	3.33	4.01	4.57	5.04

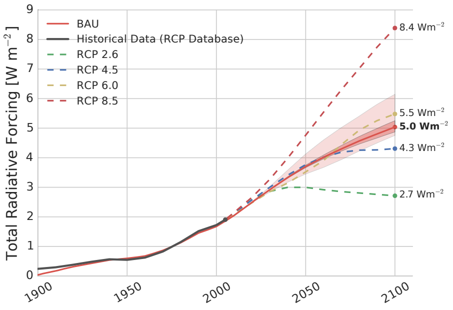

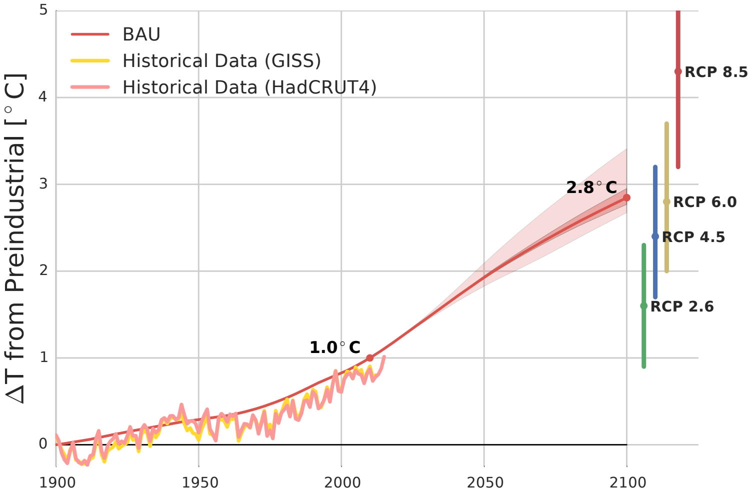

Figure S7 Clockwise from top left: table of radiative forcings due to all GHGs in the *BAU* scenario. Forcings for all non-CO2 gases are nominally equal to RCP 4.5. Total radiative forcing in *BAU* scenario compared to all RCPs. Temperature change relative to preindustrial climate (°C). Historical data drawn from GISS and HadCrut4. In the plots, the darker shaded range indicates the effects of high and low population projections, and the larger, lighter range indicates the effect of switching all non-CO2 pathways to RCP 2.6 and RCP 8.5.

Operating Expenditures (\$US kg^{-1})	
Cultivation	0.078
Dewatering	0.105
Drying	0.010
CO_2	1.050
Labor	0.264
Maintenance	0.144
Total	1.652
Amortization of capital expenditures	0.191
Total with amortization	1.843

Table S2 Marginal costs of algae production for feedstock at the Algae Energy Farm in Queensland, Australia.

of the total extent of permanent pastures & meadows; arable land & permanent crops; and forest plantations are shown for the *Alg-Feed (40%)* scenario in Fig. S9.

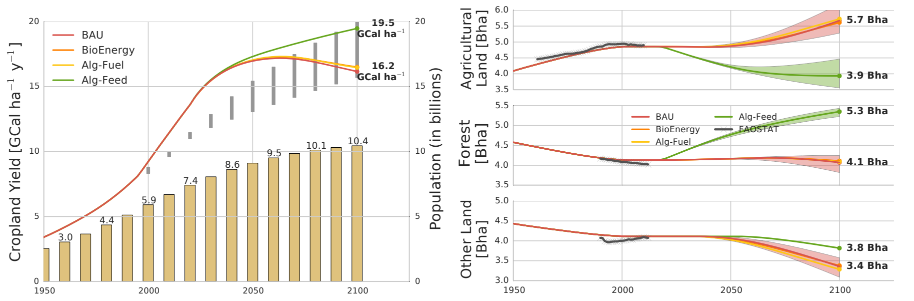

Figure S8 (L) Time series of global cropland food and feed yields (shown for all scenarios) and global population (shown for *BAU* scenario). Independent, econometric projections of crop yields projections, displayed as dark grey bars, are used to validate model yield projections [2]. (R) Global agricultural, forest, and "other" land usage in billions of hectares. "Other" includes all land not classified as agricultural, forest, or urban/industrial. Shaded ranges show the effects of population growth, the leading source of error for the *BAU* and *Alg-Feed (40%)* agricultural land projections. Historical data from FAOSTAT used for calibration [3].

3.3 Emissions

Projected annual emissions for five major fuel sources and land use & land use change (LULUC) in the *BAU*, *BioEnergy*, *Alg-Fuel* and *Alg-Feed (40%)* scenarios in 2100 are shown in Table S3. In the *Alg-Fuel* and *Alg-Feed (40%)* scenarios, total emissions are also tabulated after CCS is used to capture 25%, 50%, or 75% of annual emissions from the energy sector. Atmospheric carbon concentrations are plotted in S10.

Figure S11 displays (left) projected net annual emissions and (right) temperature anomalies in year 2100 of the FeliX simulation for a range of CCS efficiencies (gross energy sector emissions reductions) and levels of algae production (as percentages of total feed demand).

		**Projected Emissions (2100)** [PgC]									
**Scenario +**		*BAU*	*BioEnergy*	*Alg-Fuel*				*Alg-Feed*			
**Em. Mitigation (CCS)**		–	–	–	*25%*	*50%*	*75%*	–	*25%*	*50%*	*75%*
Source	(I_{Em} [gC MJ^{-1}])										
Oil	(20.0)	3.4	2.4	1.2	0.9	0.6	0.3	0.0	0.0	0.0	0.0
Gas	(15.3)	3.3	2.7	2.4	1.8	1.2	0.6	1.5	1.1	0.8	0.4
Coal	(25.8)	2.6	1.6	1.3	1.0	0.6	0.3	0.6	0.5	0.3	0.2
Solar	(1.03)	0.1	0.2	0.2	0.2	0.1	0.1	0.2	0.2	0.1	0.0
Biomass	(2.81)	0.4	0.5	0.8	-0.7	-2.3	-3.7	1.3	-1.7	-5.0	-8.0
LULUC	–	2.7	2.5	2.5	2.5	2.4	2.4	0.8	0.7	0.7	0.7
**Total**		12.5	10.0	8.4	5.7	2.7	0.1	4.5	0.9	-3.1	-6.7

Table S3 Emissions intensity and annual emissions for five major fuel sources and land use & land use change (LULUC) in the *BAU*, *BioEnergy*, *Alg-Fuel* and *Alg-Feed (40%)* scenarios in 2100. In the *Alg-Fuel* and *Alg-Feed (40%)* scenarios, total emissions are also tabulated after CCS is used to capture 25%, 50%, or 75% of annual emissions from the energy sector. Net emissions intensity is shown for biomass. Emissions intensity source: IIASA Global Energy Assessment

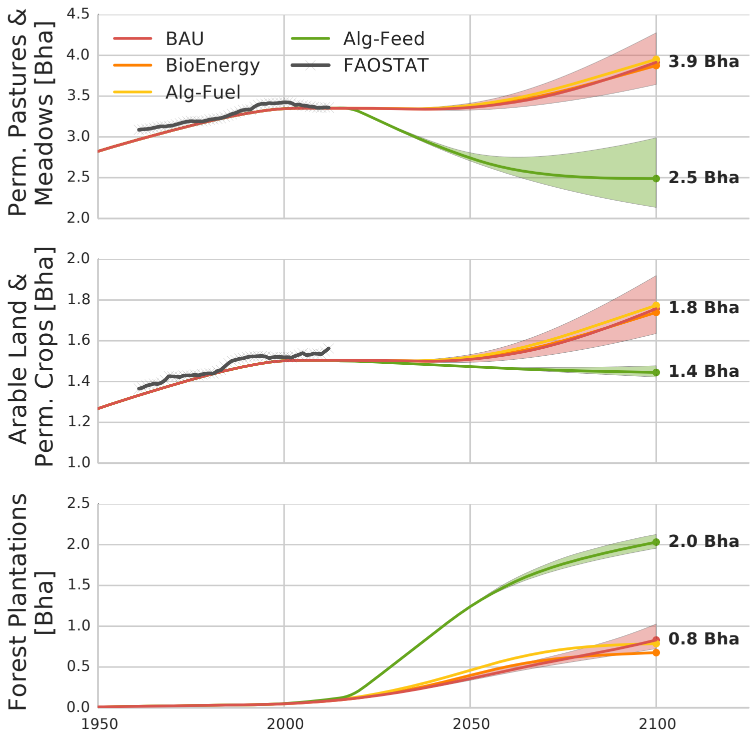

Figure S9 From top: time series of total extent of permanent pastures & meadows; arable land & permanent crops; and forest plantations. In the *Alg-Feed (40%)* scenario, microalgae is used to meet 40% of demand for feed. Of the 1.8 Bha agricultural land this frees, 1.4 Bha is converted to forest plantations. Shaded ranges show the effects of population growth, the leading source of systematic error on agricultural land use projections. Historical data from FAOSTAT used for calibration [3].

4 Sensitivity Analysis

An envelope of plausibility has been defined for eleven FeliX model parameters. In all cases, the range of this envelope is reasonably conservative, as defined relative to the RCPs, SSPs, and other similar projections. Most importantly, this range establishes the relative magnitude of the effect that each parameter has on emissions projections. The results of this sensitivity analysis on emissions savings in *Alg-Feed (40%)* relative to *BAU* are shown in Table 2 of the main paper. Absolute effects on the *BAU* and *Alg-Feed (40%)* scenarios are listed in Tab. S4.

For each parameter, two additional scenarios are defined in which the parameter is shifted above or below its nominal value (as described in *Error Analysis* subsections throughout). The impacts of each shift on total cumulative emissions [2010-2100] is calculated and shown relative to nominal values (at top) in Table S4.

Alternative per capita energy demand and population pathways have the largest effects on cumulative emissions (±8% and ±6%, respectively) in the *BAU* scenario. Emissions reductions in the *Alg-Feed (40%)* scenario are dependent on several additional factors or assumptions, including especially biomass fixed emissions and plantation productivity.

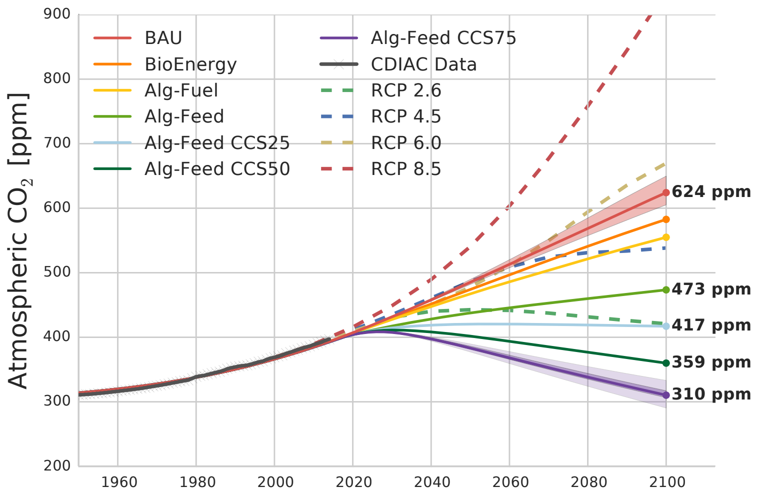

Figure S10 Time series of atmospheric CO_2 concentrations. Dark shaded ranges show the effects of population growth on the *BAU* and *Alg-Feed (40%)+CCS* projections, and the lighter range depicts sensitivity of the latter scenario to energy crop land productivity. For comparison, the four IPCC RCPs are also displayed [4]. Historical data from CDIAC used for validation [5].

	CCS Energy Sector Emissions Mitigation						
	0%	25%	35%	45%	55%	65%	75%
BAU	12.5	9.1	7.6	6.2	4.8	3.5	2.1
BioEnergy	10.0	6.9	5.5	4.3	3.0	1.9	0.6
Alg-Feed 10%	6.1	3.1	1.8	0.6	-0.7	-1.8	-3.1
Alg-Feed 20%	5.2	2.0	0.5	-0.7	-2.1	-3.3	-4.7
Alg-Feed 30%	4.8	1.4	-0.2	-1.5	-2.9	-4.3	-5.8
Alg-Feed 40%	4.5	0.9	-0.7	-2.2	-3.7	-5.1	-6.7

	CCS Energy Sector Emissions Mitigation						
	0%	25%	35%	45%	55%	65%	75%
BAU	2.8	2.6	2.4	2.3	2.2	2.1	2.0
BioEnergy	2.7	2.4	2.3	2.1	2.0	1.9	1.7
Alg-Feed 10%	2.3	2.0	1.9	1.8	1.6	1.5	1.3
Alg-Feed 20%	2.2	1.9	1.8	1.6	1.5	1.4	1.2
Alg-Feed 30%	2.2	1.8	1.7	1.6	1.4	1.3	1.1
Alg-Feed 40%	2.1	1.8	1.6	1.5	1.3	1.2	1.0

Figure S11 **(Left)** Net annual emissions from the energy and land use sectors [GtC y^{-1}] in year 2100 for a range of emissions mitigation with CCS (gross energy sector emissions reductions) and levels of algae production (as percentage of total feed demand). **(Right)** Projected temperature anomalies relative to preindustrial [°C] in year 2100 of the FeliX simulation. In both tables, global temperature change of 2.0±0.2°C is indicated by the yellow cells. Green cells indicate sub-2°C warming in year 2100 of the simulation, and red cells indicate greater than 2°C warming.

Cumulative Emissions (2011-2100) [PgC]					
		BAU		*Alg-Feed (40%)*	
		Nominal = 1099.2 PgC		Nominal = 555.2 PgC	
**Parameter**	**Nominal Value**	**Down (Rel.)**	**Up (Rel.)**	**Down (Rel.)**	**Up (Rel.)**
Agricultural Emissions	0.24 tC ha^{-1} y^{-1} †	1066.5 (-3.0%)	1131.8 (3.0%)	527.4 (-5.0%)	582.3 (4.9%)
Agricultural Residues	5.5 tDM ha^{-1} y^{-1}	–	–	578.0 (4.1%)	534.7 (-3.7%)
Agricultural Yields	11.4 GCal ha^{-1} y^{-1} †	1126.3 (2.5%)	1077.3 (-2.0%)	586.5 (5.6%)	534.7 (-3.7%)
Algae Program Start Date	2015	–	–	–	605.3 (9.0%)
Biomass Fixed Emissions	0.05 tC tDM^{-1}	1078.9 (-1.8%)	1119.4 (1.8%)	477.1 (-14.1%)	632.7 (14.0%)
Energy Crop Productivity	20 tDM $ha^{-1}y^{-1}$	1128.1 (2.6%)	1089.5 (-0.9%)	555.7 (0.0%)	552.0 (-0.6%)
Energy Demand	69 GJ cap^{-1} y^{-1} †	1011.3 (-8.0%)	1193.2 (8.5%)	495.0 (-10.8%)	624.6 (12.5%)
Feed Pct. from Algae	40%	–	–	646.4 (16.4%)	–
Food Demand (Animal)	433 kCal cap^{-1} day^{-1} †	1088.7 (-1.0%)	1108.8 (0.9%)	542.0 (-2.4%)	567.9 (2.3%)
Food Demand (Vegetal)	2432 kCal cap^{-1} day^{-1} †	1089.8 (-0.8%)	1108.0 (0.8%)	550.1 (-0.9%)	559.9 (0.9%)
Forest C Sequestration	85 tC ha^{-1}	1097.9 (-0.1%)	1100.4 (0.1%)	580.7 (4.6%)	529.0 (-4.7%)
Global GDP	$11,475 US$2005 cap^{-1} y^{-1} †	1099.2 (0.0%)	1097.8 (-0.0%)	561.8 (1.2%)	549.1 (-1.1%)
Non-CO_2 Emissions	RCP 4.5	1098.8 (0.0%)	1099.9 (0.1%)	554.7 (0.0%)	555.3 (0.0%)
Plantation Productivity	10 tDM ha^{-1} y^{-1}	1105.8 (-0.2%)	1099.7 (0.0%)	599.0 (7.9%)	521.5 (-6.1%)
World Population	UNDESA Med. Variant [26]	1037.5 (-5.6%)	1182.6 (7.6%)	507.1 (-8.7%)	628.4 (13.2%)

† Denotes time-dependent variable. Nominal value in 2010 is listed.

Table S4 Sensitivity analysis of leading model parameters affecting cumulative (2010-2100) emissions projections in the *BAU* and *Alg-Feed (40%)* scenarios. Absolute shifts listed in this table are used to calculate relative effects in Table 2 in the main paper. Parameters are shifted as described in relevant sections of this document.

5 Author Information, Acknowledgments, and References

Competing interests

The authors declare that they have no competing interests.

Author's contributions

BW and FR established the structure and scope of the FeliX model. AS and MO generated the foundational ideal for the analysis. BW, MO, FR, and MH defined and developed the scenarios presented. BW and AP contributed to literature review and projections (RCPs, SSPs, and agricultural yields). PS, MH, and AS consulted on algaculture cost, productivity, and other technical parameters. BW, FR, MO, PS, AP, MH, IJ, PC, AS, JP, PH, and FK contributed to the preparation and validation of the BAU scenario, set the scope of the analysis, and edited the manuscript.

Acknowledgements

The authors acknowledge support from the European Research Council Synergy grant ERC-2013-SyG-610028 IMBALANCE-P.

FeliX is a model that has been developed and applied in a team effort at IIASA over the course of several years and projects, led originally by ESM staff member Dr. Felicjan Rydzak, and with significant foundational contributions from ESM staff members Dr. Michael Obersteiner, Florian Kraxner, Dr. Steffen Fritz, and Dr. Ian McCallum.

Author details

[1]Ecosystems Services and Management, International Institute for Applied Systems Analysis, Schlossplatz 1, Laxenburg, Austria. [2]Commonwealth Scientific and Industrial Research Organisation, Brisbane, Australia. [3]Algae Biotechnology Laboratory, School of Agriculture and Food Sciences, The University of Queensland, Brisbane, Australia. [4]Laboratoire des Sciences du Climat et de L'Environnement, CEA-CNRS-UVSQ, Gif-sur-Yvette, France. [5]University of Antwerp, Wilrijk, Belgium. [6]CSIC, Global Ecology Unit CREAF-CSIC-UAB, Cerdanyola del Valles (Catalonia), Spain. [7]CREAF, Cerdanyola del Valles (Catalonia), Spain.

References

1. Global Energy Assessment: Toward a sustainable future. Technical report, IIASA, Laxenburg, Austria (2012)
2. Herrero, M., Havlik, P., McIntire, J., Palazzo, A., Valin, H.: African livestock futures: Realizing the potential of livestock for food security, poverty reduction and the environment in sub-saharan africa. Technical report, Office of the Special Representative of the UN Secretary General for Food Security and Nutrition and the United Nations System Influenza Coordination (UNSIC) (2014)
3. Food and Agriculture Organization of the United Nations: Food and Agriculture Organization of the United Nations, FAOSTAT Database. <http://faostat3.fao.org/>
4. Vuuren, D.P., et al: The representative concentration pathways: an overview. Clim. Change **109**, 5–31 (2011)
5. Boden, T.A., et al: Global, regional, and national fossil-fuel CO_2 emissions. Technical report, Carbon Dioxide Information Analysis Center, Oak Ridge National Laboratory, U.S. Department of Energy (2013)
6. Forrester, J.W.: Industrial dynamics: a major breakthrough for decision makers. Harvard business review **36**(4), 37–66 (1958)
7. Forrester, J.W.: Industrial dynamics. Journal of the Operational Research Society **48**(10), 1037–1041 (1997)
8. Sterman, J.D.: Business Dynamics: Systems Thinking and Modeling for a Complex World vol. 19. Irwin/McGraw-Hill, Boston (2000)
9. Richardson, G.P., Pugh III, A.I.: Introduction to System Dynamics Modeling with DYNAMO. Productivity Press Inc., New York (1981)
10. Lyneis, J.M.: Corporate Planning and Policy Design: A System Dynamics Approach. MIT Press, Cambridge, MA (1980)
11. Oeschger, H., Siegenthaler, U., Schotterer, U., Gugelmann, A.: A box diffusion model to study the carbon dioxide exchange in nature. Tellus **27**(2), 168–192 (1975)
12. Goudriaan, J., Ketner, P.: A simulation study for the global carbon cycle, including man's impact on the biosphere. Climatic Change **6**(2), 167–192 (1984)
13. Bolin, B.: Requirements for a satisfactory model of the global carbon cycle and current status of modeling efforts. In: The Changing Carbon Cycle, pp. 403–424. Springer, Heidelberg (1986)
14. Rotmans, J.: An Integrated Model to Assess the Greenhouse Effect: An Integrated Model to Assess the Greenhouse Effect vol. 1. Springer, Heidelberg (1990)
15. Nordhaus, W.D.: Managing the Global Commons: the Economics of Climate Change vol. 31. MIT Press, Cambridge, MA (1994)
16. Fiddaman, T.S.: Feedback complexity in integrated climate-economy models. PhD thesis, Massachusetts Institute of Technology (1997)
17. Richardson, G.P., Andersen, D.F.: Teamwork in group model building. System Dynamics Review **11**(2), 113–137 (1995)
18. Vennix, J.A.: Group Model Building: Facilitating Team Learning Using System Dynamics vol. 2001. Wiley, Chichester (1996)
19. Andersen, D.F., Richardson, G.P., Vennix, J.A.: Group model building: adding more science to the craft. System dynamics review **13**(2), 187–201 (1997)
20. Sterman, J.D., Richardson, G.P.: An experiment to evaluate methods for estimating fossil fuel resources. Journal of forecasting **4**(2), 197–226 (1985)
21. Schellnhuber, H.J.: Tipping elements in the earth system. Proc. Natl Acad. Sci. USA **106**(49), 20561–20563 (2009)
22. Steffen, W., Crutzen, P.J., McNeill, J.R.: The anthropocene: are humans now overwhelming the great forces of nature. AMBIO: A Journal of the Human Environment **36**(8), 614–621 (2007)
23. Sterman, J.D.: Appropriate summary statistics for evaluating the historical fit of system dynamics models. Dynamica **10**, 51–66 (1984)

24. Rydzak, F., Obersteiner, M., Kraxner, F., Fritz, S., McCallum, I.: FeliX3 - Impact Assessment Model. Technical report, International Institute for Applied Systems Analysis (2013). Available for download at www.felixmodel.org
25. Walsh, B.: FeliX Model. www.felixmodel.org. Accessed 1 May 2015.
26. Gerland, P., et al.: World population stabilization unlikely this century. Science **346**(6206), 234–237 (2014)
27. O'Neill, B., Kriegler, E., Riahi, K., Ebi, K., Hallegatte, S., Carter, T., Mathur, R., van Vuuren, D.P.: A new scenario framework for climate change research: the concept of shared socioeconomic pathways. Climatic Change **122**(3), 387–400 (2014)
28. Maddison, A.: Historical statistics of the world economy, 1-2006 AD. Groningen Growth and Development Centre (2006)
29. Herrero, M., Havlik, P., McIntire, J., Palazzo, A., Valin, H.: African livestock futures: Realizing the potential of livestock for food security, poverty reduction and the environment in sub-saharan africa (2014)
30. Sterman, J., Fiddaman, T., Franck, T., Jones, A., McCauley, S., Rice, P., Sawin, E., Siegel, L.: Climate interactive: the C-ROADS climate policy model. System Dynamics Review **28**(3), 295–305 (2012)
31. Fiddaman, T.S.: Exploring policy options with a behavioral climate–economy model. System Dynamics Review **18**(2), 243–267 (2002)
32. Nordhaus, W.D.: The DICE model: Background and structure of a dynamic integrated climate-economy model of the economics of global warming. Technical report, Cowles Foundation for Research in Economics, Yale University (1992)
33. Nordhaus, W.D.: Managing the Global Commons: the Economics of Climate Change vol. 31. MIT Press, Cambridge, MA (1994)
